# Iso-α-Acids, the Bitter Components of Beer, Suppress Microglial Inflammation in rTg4510 Tauopathy

**DOI:** 10.3390/molecules23123133

**Published:** 2018-11-29

**Authors:** Yasuhisa Ano, Yuta Takaichi, Kazuyuki Uchida, Keiji Kondo, Hiroyuki Nakayama, Akihiko Takashima

**Affiliations:** 1 Graduate School of Agricultural and Life Sciences, The University of Tokyo, Tokyo 113-8657, Japan; takaichi.yuta.5110@gmail.com (Y.T.); auchidak@mail.ecc.u-tokyo.ac.jp (K.U.); anakaya@mail.ecc.u-tokyo.ac.jp (H.N.); 2 Research Laboratories for Health Science & Food Technologies, Kirin Company Ltd., Kanagawa 236-0004, Japan; kondok@kirin.co.jp; 3 Department of Aging Neurobiology, National Center for Geriatrics and Gerontology, Obu 474-8511, Japan; 20160021@gakushuin.ac.jp; 4 Faculty of Science, Gakushuin University, Tokyo 171-8588, Japan

**Keywords:** inflammation, iso-α-acids, microglia, tau, tauopathy

## Abstract

Due to the growth in aging populations, prevention for cognitive decline and dementia are in great demand. We previously demonstrated that the consumption of iso-α-acids (IAA), the hop-derived bitter compounds in beer, prevents inflammation and Alzheimer’s disease pathology in model mice. However, the effects of iso-α-acids on inflammation induced by other agents aside from amyloid β have not been investigated. In this study, we demonstrated that the consumption of iso-α-acids suppressed microglial inflammation in the frontal cortex of rTg4510 tauopathy mice. In addition, the levels of inflammatory cytokines and chemokines, including IL-1β and MIP-1β, in the frontal cortex of rTg4510 mice were greater than those of wild-type mice, and were reduced in rTg4510 mice fed with iso-α-acids. Flow cytometry analysis demonstrated that the expression of cells producing CD86, CD68, TSPO, MIP-1α, TNF-α, and IL-1β in microglia was increased in rTg4510 mice compared with wild-type mice. Furthermore, the expression of CD86- and MIP-1α-producing cells was reduced in rTg4510 mice administered with iso-α-acids. Moreover, the consumption of iso-α-acids reduced the levels of phosphorylated tau in the frontal cortex. Collectively, these results suggest that the consumption of iso-α-acids prevents the inflammation induced in tauopathy mice. Thus, iso-α-acids may help in preventing inflammation-related brain disorders.

## 1. Introduction

Dementia and cognitive impairment are becoming an increasing burden not only on patients and their families, but also on national healthcare systems worldwide, concomitant with the rapid growth in aging populations. Owing to the lack of disease-modifying therapies for dementia, preventive approaches, including diet, exercise, and learning are garnering increased attention. Etiological studies of lifestyle have demonstrated that low-to-moderate consumption of alcohol, such as wine and beer, may reduce the risk of cognitive decline and the development of dementia. Indeed, individuals who consume low-to-moderate levels of alcoholic beverages on a daily basis were shown to have a significantly lower risk of developing a neurodegenerative disease, as compared with individuals who abstained from alcohol beverages or drank heavily [1,2,3]. Apart from the effects of alcohol itself, resveratrol, a polyphenolic compound present in red wine, has been shown to be neuroprotective [4,5,6,7]. We previously demonstrated that the consumption of iso-α-acids, the bitter components present in beer, prevents Alzheimer’s pathology in 5 × FAD transgenic model mice. In addition, iso-α-acids suppress the microglial inflammation induced by amyloid β deposition in the brain, resulting in protection against cognitive decline. Iso-α-acids activate the peroxisome proliferator-activated receptor-γ (PPAR-γ) and regulate microglial phagocytosis and inflammation [8]. However, the effects of iso-α-acids on inflammation induced by other agents aside from amyloid β have not been investigated. In Alzheimer’s disease, neurofibrillary tangles (NFTs) composed of hyperphosphorylated tau are observed in each brain region with aging, as well as senile plaques composed of amyloid β [9]. Tauopathy is characterized by fibrillar tau accumulation in neurons and glial cells, which is associated with neuronal dysfunction [10]. Proliferation and activation of microglia in the brain around NFTs and senile plaques are prominent features of Alzheimer’s disease. Inflammation caused by activated microglia is associated with the disease progressions [11]. rTg4510 tauopathy model mice overexpress human P301L mutant tau and show neuroinflammation in the brain, accompanied by disease progression [12]. On the other hand, there is no report evaluating the preventive effects of nutritional components with anti-inflammatory activity in rTg4510 mice. Therefore, in the present study, the effects of iso-α-acids on inflammation in rTg4510 tauopathy mice were investigated.

## 2. Results

### 2.1. Effects of Iso-α-Acids on Inflammation in the Brain with Tauopathy

**Figure 1:**
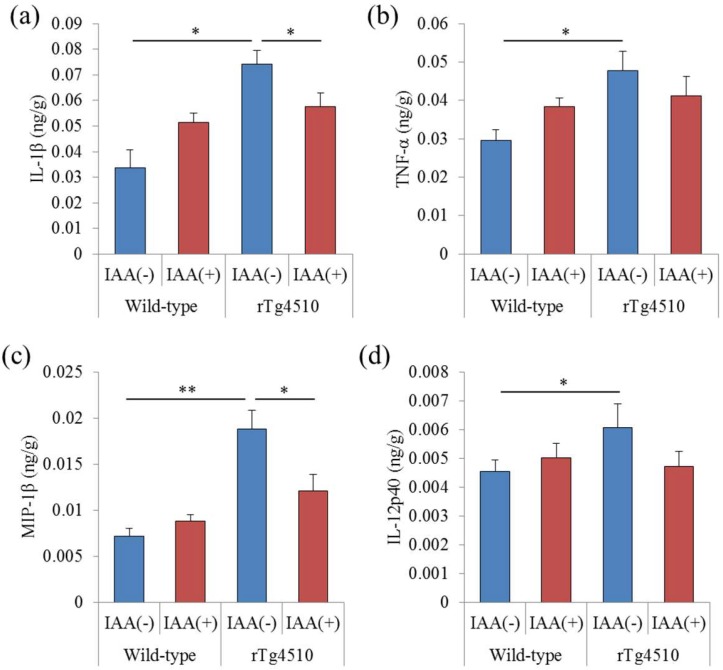
Effects of iso-α-acids on rTg4510 mice. Three-month-old rTg4510 mice and wild-type mice were fed 0% or 0.05% (*w/w*) iso-α-acids (IAA) for three months. (**a**–**d**), the levels of IL-1β, TNF-α, MIP-1α, and IL-12p40 in the frontal cortex, respectively. Data are the means ± SEM of 12 (wild-type mice without IAA), 6 (wild-type mice with IAA), 12 (rTg4510 mice without IAA), and 10 (rTg4510 mice with IAA) mice. *p*-values shown in the graph were calculated by one-way ANOVA, followed by the Tukey-Kramer test. * *p* < 0.05 and ** *p* < 0.01.

To evaluate the effects of iso-α-acids on inflammation in the brain of rTg4510 tauopathy mice, the levels of proinflammatory cytokines and chemokines in the frontal cortex of tauopathy mice fed with iso-α-acids were measured. The levels of IL-1β, TNF-α, MIP-1β, and IL-12p40 in the frontal cortex of rTg4510 mice were higher than those of wild-type mice (Figure 1a–d). The administration of iso-α-acids reduced the levels of IL-1β and MIP-1β in rTg4510 mice (Figure 1a,c), but did not change those in wild-type mice. These results indicate that proinflammatory cytokines and chemokines are increased in the frontal cortex in rTg4510 mice, and the consumption of iso-α-acids reduce the inflammation induced in tauopathy mice.

### 2.2 Effects of Iso-α-Acids on Microglial Phenotype in the Brain of Tauopathy

**Figure 2:**
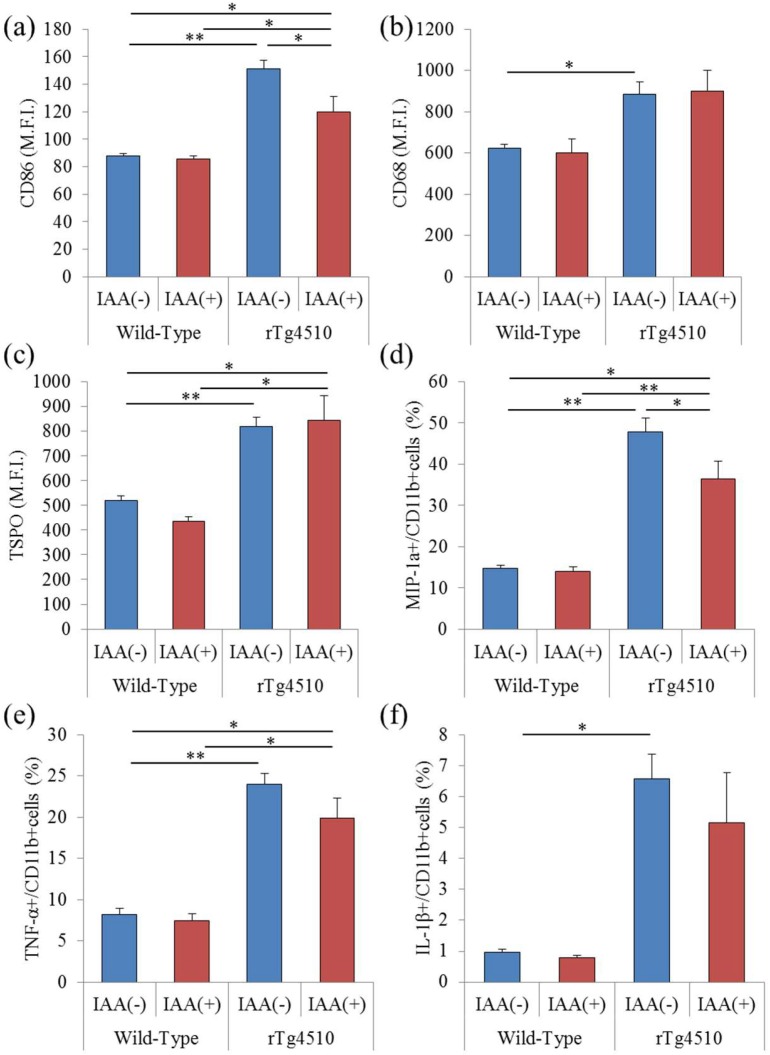
Analysis of microglia in rTg4510 mice. Microglia in the brains of rTg4510 mice were isolated using magnetic cell sorting and analyzed using a flow cytometer. (**a**–**c**), the expression of CD86, CD68, and TSPO in CD11b-positive microglia of rTg4510 or wild-type mice, respectively. (**d**–**f**), the percentages of intracellular MIP-1α-, TNF-α-, and IL-1β-producing cells in CD11b-positive cells, respectively. Data are the means ± SEM of five mice in each group. *p*-values shown in the graph were calculated by one-way ANOVA, followed by the Tukey-Kramer test. * *p* < 0.05 and ** *p* < 0.01.

To evaluate the effects of iso-α-acids on microglia in rTg4510 tauopathy mice, CD11b-positive microglia were isolated and analyzed using flow cytometry. The expression of CD86, a costimulatory molecule, on CD11b-positive cells in the brain was increased in rTg4510 mice compared with wild-type mice and reduced in rTg4510 mice fed with iso-α-acids (Figure 2a). The expressions of CD68 and TSPO in CD11b-positive microglia were increased in rTg4510 mice compared with wild-type, but did not change in rTg4510 mice fed with iso-α-acids (Figure 2b,c). MIP-1α-, TNF-α-, and IL-1β-producing cells were also increased in rTg4510 mice, and MIP-1α-producing cells were decreased by iso-α-acids (Figure 2d–f). These results indicate that the microglia phenotype was induced into the proinflammatory type in rTg4510 mice, and some of these inflammatory inductions were suppressed by the consumption of iso-α-acids.

### 2.3. Effects of Iso-α-Acids on Tau Phosphorylation in Tauopathy Mice

**Figure 3:**
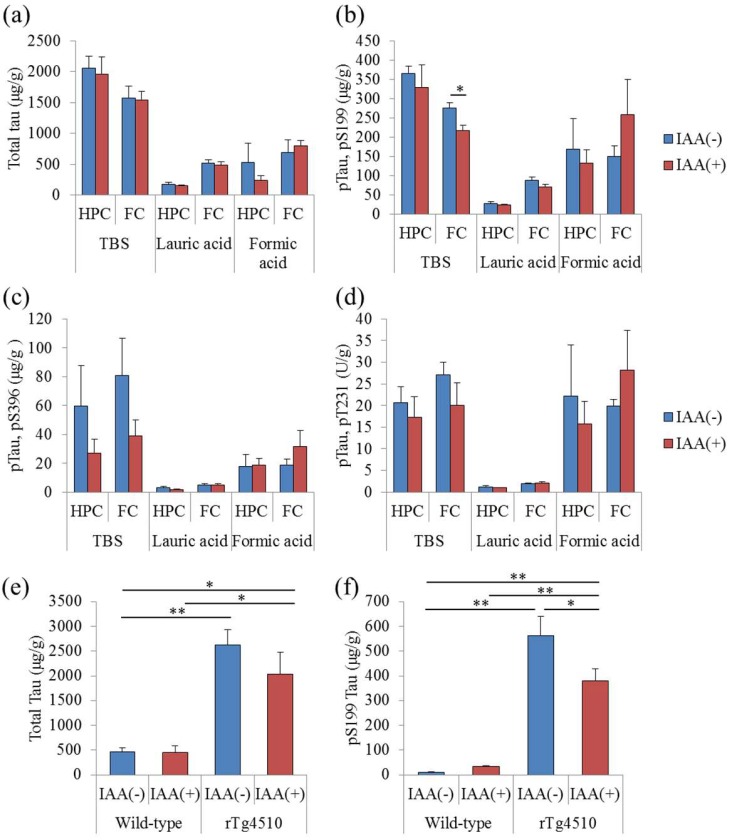
Effects of iso-α-acids on the phosphorylation of tau in rTg4510 mice. Three-month-old rTg4510 mice and wild-type mice were fed 0% or 0.05% (*w/w*) iso-α-acids (IAA) for three months. (**a**–**d**), the levels of total tau and phosphorylated tau (pS199, pS396, and pT231) soluble in tris-buffered saline (TBS) buffer, lauric acid, and formic acid of rTg4510 were measured. *p*-values shown in the graph were calculated by Student’s *t*-test. * *p* < 0.05. (**e**,**f**), the levels of total tau and phosphorylated tau (pS199) soluble in TBS buffer of rTg4510 and wild-type mice fed with 0% or 0.05% (*w/w*) IAA, respectively. *p*-values shown in the graph were calculated by one-way ANOVA, followed by the Tukey-Kramer test. * *p* < 0.05 and ** *p* < 0.01.

To evaluate the effects of iso-α-acids on the phosphorylation of tau, the levels of total tau and phosphorylated tau in the hippocampus and frontal cortex were measured. The levels of total tau were not changed by the consumption of iso-α-acids (Figure 3a). However, the levels of phosphorylated tau (pS199) soluble in TBS buffer in the frontal cortex were significantly decreased with the consumption of iso-α-acids (Figure 3b). The levels of phosphorylated tau (pS396 and pT231) in rTg4510 mice fed with iso-α-acids were lower, but this change was not significantly different from control rTg4510 mice (Figure 3c,d). pTau soluble in lauric acid and formic acid was not changed by the administration of iso-α-acids. The levels of phosphorylated tau in the hippocampus of rTg4510 mice and the levels of total tau and phosphorylated tau (pS199) in the frontal cortex of wild-type mice were not changed by the consumption of iso-α-acids. These results indicate that the consumption of iso-α-acids reduces the phosphorylation of tau in rTg4510 mice.

## 3. Discussion

In the present study, we demonstrated that the consumption of iso-α-acids mitigated microglial inflammation and the phosphorylation of tau in the frontal cortex of tauopathy rTg4510 mice. To evaluate the effects of iso-α-acids on tauopathy, rTg4510 tauopathy mice were fed with iso-α-acids, and the levels of proinflammatory cytokines and pTau were measured. The consumption of iso-α-acids suppressed the levels of cytokines and chemokines in the frontal cortex and induced microglia into the anti-inflammatory type in rTg4510 mice. We previously reported that iso-α-acids activate PPAR-γ [13], and PPAR-γ activation by iso-α-acids is involved in the suppression of microglial inflammation using primary microglia [8]. It has also been reported that PPAR-γ activation induces microglia into the M2 anti-inflammatory type [14,15]. Pioglitazone, an agonist of PPAR-γ, induced microglia into the M2 type and showed anti-inflammatory effects in vivo [16]. These results suggest that iso-α-acids also suppress tau-induced microglial inflammation.

Inflammation in the brain has increasingly become a focus for studies of preventive and therapeutic approaches for Alzheimer’s disease [17]. Epidemiological investigations have suggested that the intake of non-steroidal anti-inflammatory drugs (NSAIDs) has a preventive effect on Alzheimer’s disease [18,19]. In addition, the potential of pioglitazone for medical treatments related to Alzheimer’s disease has been suggested. Microglia play a crucial role in inflammation in the brain. In general, microglia remove the old synapses and waste products in the brain to maintain the environment [20]. On the other hand, massively activated microglia produce neurotoxic substances, including reactive oxygen spices and inflammatory cytokines [21]. It has been suggested that the polarization of microglia between the M1 inflammatory type and the M2 anti-inflammatory type is important for improving the neurological pathology and cognitive decline observed in Alzheimer’s disease [22]. CD86 and MIP-1 proinflammatory markers of microglia are increased in rTg4510 mice [23,24], and consumption of iso-α-acids suppressed the expression of CD86 in rTg4510 mice. Therefore, it is suggested that iso-α-acids suppress the induction of microglia into the M1 proinflammatory type in rTg4510 mice.

We next evaluated the effects of iso-α-acids on the phosphorylation of tau, and the concentrations of tau and pTau were measured. pTau (Ser199), soluble in TBS, in rTg4510 mice fed with iso-α-acids was significantly lower than that of control rTg4510 mice. It has been reported that inflammation in the brain accelerates the phosphorylation of tau [25,26]. Intraventricular injection with lipopolysaccharide (LPS) in rTg4510 mice induces microglia into the proinflammatory type and increases the phosphorylation of tau (Ser199) in the frontal cortex and hippocampus [25]. These reports suggest that the suppression of inflammation in the frontal cortex of rTg4510 mice reduces the phosphorylation of tau. However, in the present study, we did not evaluate the direct effects of iso-α-acids on the phosphorylation of tau. Thus, further study is needed to further evaluate the relationship between inflammation and phosphorylation of tau.

In summary, in the present study, we analyzed the microglial inflammatory phenotype in rTg4510 mice; induced microglial inflammation in rTg4510 was suppressed by the consumption of iso-α-acids. Iso-α-acids are considered a safe food material because they are generated from α-acids in hops, and hops have been used as a material for brewing beer for more than 1000 years. The consumption of iso-α-acids suppresses the inflammation induced by various agents, including amyloid β [8], a high-fat diet [27], and pTau; therefore, it may help in preventing inflammatory-related brain disorders. Further study as part of a clinical trial is needed to evaluate the effects of iso-α-acids on cognitive function.

## 4. Materials and Methods

### 4.1. Animals

rTg4510 mice [28], a transgenic mouse model for human tauopathy, and control FVB/N-C57BL/6J mice (wild-type mice) were used in this study. Animals were maintained in an experimental facility at the University of Tokyo. rTg4510 mice overexpress human tau that contains the frontotemporal dementia-associated P301L mutation, and tau expression can be suppressed with doxycycline treatment [28]. For the expression of mutant tau in rTg4510 mice, the mutated gene, located downstream of a tetracycline-operon-responsive element, must be co-expressed with an activator transgene consisting of a tet-off open reading frame located downstream of the Ca^2+^-calmodulin kinase II promoter elements [28]. Wild-type control mice lack both the tau responder and the activator transgene. Mice under 3 months of age were housed in cages, with free access to a standard purified rodent growth diet (AIN-93G, Oriental Yeast, Tokyo, Japan); mice over 3 months of age were housed with free access to a maintenance diet (AIN-93M, Oriental Yeast). Three-month-old mice were fed 0% or 0.05% (*w/w*) iso-α-acids for 3 months. The number of mice were 12 (wild-type mice without iso-α-acids), 6 (wild-type mice with iso-α-acids), 12 (rTg4510 mice without iso-α-acids), and 10 (rTg4510 mice with iso-α-acids). The Institutional Animal Care and Use Committee of the Graduate School of Agricultural and Life Science at the University of Tokyo approved all experiments in 2017 (Approval No. P17-020). All efforts were made to minimize suffering.

### 4.2. Preparation of Iso-α-Acids

**Figure 4:**
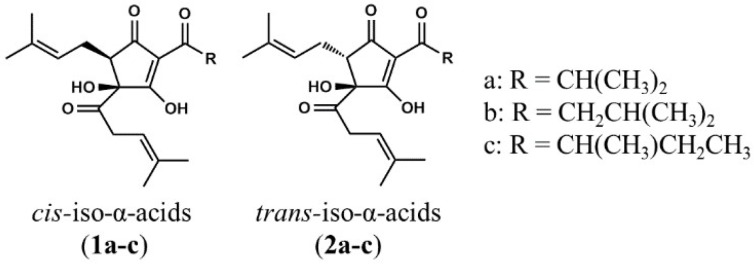
Chemical structures of iso-α-acids. Structures of *cis*-iso-α-acids: *Cis*-isocohumulone (**1a**), *cis*-isohumulone (**1b**)**,** and *cis*-isoadhumulone (**1c**). Structures of *trans*-iso-α-acids: *Trans*-isocohumulone (**2a**), *trans*-isohumulone (**2b**), and *trans*-isoadhumulone (**2c**).

Iso-α-Acids consist predominantly of three congeners: Cohumulone, humulone, and adhumulone. During the brewing process, they are each isomerized into two epimeric isomers, namely, *cis*- and *trans*-iso-α-acids (Figure 4). A purchased isomerized hop extract (IHE; Hopsteiner, Mainburg, Germany) with 30.5% (*w/v*) iso-α-acids, comprising *cis*-isocohumulone (7.61% *w/v*), *cis*-isohumulone (14.0% *w/v*)**,** and *cis*-isoadhumulone (3.37% *w/v*), *trans*-isocohumulone (1.74% *w/v*), *trans*-isohumulone (3.05% *w/v*), and *trans*-isoadhumulone (0.737% *w/v*)was used in this study.

### 4.3. Microglia Analysis

Primary microglial cells were isolated from the brain via magnetic cell sorting after conjugation with anti-CD11b antibodies (Miltenyi Biotec, Bergisch Gladbach, Germany), as previously described [29]. Isolated CD11b-positive cells (>90% pure as evaluated by flow cytometry) were stained with anti-CD86-FITC (eBioscience, CA, USA), anti-CD68-APC (BioLegend, CA, USA), anti-TSPO-PE (PBR, Abcam, Cambridge, UK), and anti-CD11b-APC-Cy7 (BD Biosciences, CA, USA) antibodies after treatment with the BD Cytofix/Cytoperm Fixation/Permeabilization kit (BD Biosciences). To measure intracellular cytokines, microglia were plated in poly-d-lysine (PDL)-coated 96-well plates (BD Biosciences) and cultured in DMEM/F-12 (Gibco, CA, USA) medium supplemented with 10% fetal calf serum (Gibco) and 100 U/mL penicillium/streptomycin (Sigma-Aldrich, MO, USA) containing a leukocyte activation-cocktail with BD GolgiPlug^TM^ (BD Biosciences) for 12 h. Microglia cells were treated with the BD Cytofix/Cytoperm Fixation/Permeabilization kit (BD Biosciences) and then stained with anti-IL-1β-FITC (eBiosciences), anti-MIP-1α-PE (eBiosciences), anti-TNF-α-APC (BD Pharmingen, CA, USA), and anti-CD11b-APC-Cy7 (BD Biosciences) antibodies. Stained cells were analyzed using a flow cytometer (FACSCanto II; BD Biosciences).

### 4.4. Cytokines and Tau Measurement in Transgenic Mice

To measure cytokines and tau in the brain, the hippocampus and frontal cortex were homogenized in TBS buffer (Wako, Monza, Monza and Brianza, Lombardy, Italy) containing protease inhibitor cocktail (Biovision, CA, USA) and phosphatase inhibitor cocktail l and II (Wako) with a multi-bead shocker (Yasui Kikai, Osaka, Japan). After centrifugation at 50,000× *g* for 20 min (MX-107, Tommy, Tokyo, Japan), the supernatant was collected. The pellets were sonicated in sarkosyl solution (1% *N*-lauroylsarcosine (Sarkosyl) in 1 mM Tris, 1 mM EGTA, 1 mM DTT, and 10% sucrose, pH 7.5), and the supernatant was collected after centrifugation at 386,000× *g* for 20 min at 4 °C. The pellets were treated with formic acid and dried. The samples were dissolved in an assay buffer (0.2 g/L KCl, 0.2 g/L KH_2_PO_4_, 8.0 g/L NaCl, 1.150 g/L Na_2_HPO_4_, 5% BSA, 0.03% Tween 20, and 1× protease inhibitor cocktail (Calbiochem) in ultrapure water, pH 7.4). The total protein concentration of each supernatant was measured using a BCA protein assay kit (ThermoScientific, Yokohama, Japan). Each supernatant was assayed for quantifying total tau and phosphorylated tau (pTau) of pS199, pS396, and pT231 (Thermo Scientific, Waltham, MA, USA) by ELISA. For quantifying cytokines and chemokines, the first supernatant was evaluated by a Bio-Plex assay system (Bio-Rad, Hercules, CA, USA).

### 4.5. Statistical Analysis

The data represent the mean ± SEM. Data were analyzed by one-way ANOVA, followed by the Tukey-Kramer test or Student’s *t*-test, as described in the figure legends. All statistical analyses were performed using the Ekuseru-Toukei 2012 software program (Social Survey Research Information, Tokyo, Japan). A value of *p* < 0.05 was considered statistically significant.
